# Structural characterization of the virulence factor nuclease A from *Streptococcus agalactiae*

**DOI:** 10.1107/S1399004714019725

**Published:** 2014-10-23

**Authors:** Andrea F. Moon, Philippe Gaudu, Lars C. Pedersen

**Affiliations:** a Laboratory of Structural Biology, National Institute of Environmental Health Sciences, National Institutes of Health, Research Triangle Park, NC 27709, USA; b INRA, UMR1319 Micalis, Domaine de Vilvert, Jouy-en-Josas, France; AgroParisTech, UMR Micalis, Jouy-en-Josas, France

**Keywords:** nuclease A, virulence factors, *Streptococcus agalactiae*

## Abstract

The group B pathogen *Streptococcus agalactiae* commonly populates the human gut and urogenital tract, and is a major cause of infection-based mortality in neonatal infants and in elderly or immunocompromised adults. Nuclease A (GBS_NucA), a secreted DNA/RNA nuclease, serves as a virulence factor for *S. agalactiae*, facilitating bacterial evasion of the human innate immune response. GBS_NucA efficiently degrades the DNA matrix component of neutrophil extracellular traps (NETs), which attempt to kill and clear invading bacteria during the early stages of infection. In order to better understand the mechanisms of DNA substrate binding and catalysis of GBS_NucA, the high-resolution structure of a catalytically inactive mutant (H148G) was solved by X-ray crystallography. Several mutants on the surface of GBS_NucA which might influence DNA substrate binding and catalysis were generated and evaluated using an imidazole chemical rescue technique. While several of these mutants severely inhibited nuclease activity, two mutants (K146R and Q183A) exhibited significantly increased activity. These structural and biochemical studies have greatly increased our understanding of the mechanism of action of GBS_NucA in bacterial virulence and may serve as a foundation for the structure-based drug design of antibacterial compounds targeted to *S. agalactiae*.

## Abstract 2

Nuclease A (NucA) is an extracellular nuclease secreted from *S. agalactiae* and is required for full virulence during infection. Crystal structures and biochemical characterization of NucA mutants reveal possible roles for surface residues in DNA substrate binding and catalysis. These results may serve as a foundation for the design of targeted antibacterial therapeutic compounds.

## 1. Introduction   

Group B *Streptococcus agalactiae* (GBS) is a Gram-positive encapsulated bacterium that commonly colonizes the human urogenital tract, asymptomatically in healthy adults (McKenna & Iams, 1998 ▶). GBS is a major cause of opportunistic infection in neonates (Doran & Nizet, 2004 ▶), the immunocompromised (Persson *et al.*, 2004 ▶) and in elderly adults (Maisey *et al.*, 2008 ▶). Infection may occur *in utero* as a result of bacterial penetration of the placental membrane or as a consequence of accidental fetal aspiration of infected vaginal secretions during labour (Allardice *et al.*, 1982 ▶). In elderly adults, *S. agalactiae* is the causative agent of nonfocused bacteraemia, endocarditis and bone or joint infections (Tazi *et al.*, 2011 ▶). Although *S. agalactiae* invasive infection is normally treated with antibiotics to ensure a favorable outcome, severe infections can lead to sepsis, meningitis and death, especially in neonates with immature immune function (Oh, 2013 ▶).

GBS has evolved a formidable array of virulence factors to aid invasion, establishment of infection and evasion of the host’s immune system (Doran & Nizet, 2004 ▶). One key component of the mammalian innate immune response is the generation of neutrophil extracellular traps (NETs), which are extracellular extrusions of a dense, fibrous matrix comprised of DNA and antimicrobial proteins (Brinkmann *et al.*, 2004 ▶). These NETs physically ensnare the invading bacteria, slowing their migration within the host and leaving them more vulnerable to immune clearance (Brinkmann & Zychlinsky, 2007 ▶). Many bacteria have evolved different membrane-associated or secreted nucleases that degrade NETs and perpetuate infection (Beiter *et al.*, 2006 ▶; Berends *et al.*, 2010 ▶; Seper *et al.*, 2013 ▶). One such nuclease, NucA (Gbs0661) from *S. agalactiae* (henceforth referred to as GBS_NucA), has been shown to degrade NETs and to improve infection persistence in the host. Animals inoculated with *S. agalactiae* expressing wild-type GBS_NucA exhibited increased bacterial proliferation and 35% higher mortality compared with those inoculated with *S. agalactiae* expressing a nuclease-inactive mutant (H148A) (Derré-Bobillot *et al.*, 2013 ▶).

GBS_NucA contains a putative N-terminal transmembrane domain which is thought to play a role in protein export. Protease removal of this transmembrane domain could result in the secretion of GBS_NucA into the extracellular environment (Derré-Bobillot *et al.*, 2013 ▶). GBS_NucA is thought to be a sequence-nonspecific DNA/RNA nuclease with endonucleolytic and exonucleolytic activity. Sequence analysis revealed that GBS_NucA displays a high sequence identity to nucleases containing a DRGH (aspartate–arginine–glycine–histidine) active-site motif, which include *S. pneumoniae* EndA, *Anabaena* sp. NucA and *Serratia marcescens* NucA (52, 34 and 28% sequence identity, respectively; Meiss *et al.*, 1998 ▶; Midon *et al.*, 2011 ▶; Shlyapnikov *et al.*, 2000 ▶). Although this family of nucleases share a common ββα metal-finger catalytic core and reaction mechanism, their global structures, localization and regulatory mechanisms can vary widely (Ghosh *et al.*, 2007 ▶; Loll *et al.*, 2009 ▶; Moon *et al.*, 2011 ▶).

Owing to the potency of GBS_NucA as a virulence factor for *S. agalactiae*, this nuclease could represent a valuable target for the development of novel antibacterial therapeutics. As a starting point for such development, we have determined high-resolution X-ray crystal structures of GBS_NucA with and without bound magnesium ion and compared them with those of other ββα metal-finger nucleases. These structural analyses provided a foundation for the biochemical exploration of surface residues that are likely to play key roles in DNA substrate binding and catalysis. Alanine substitution of several residues surrounding the catalytic center (R111A, R116A/R117A, H142A, N179A, Q180A and R197A/K197A) resulted in a profound inhibition of nuclease activity. Conversely, the mutagenesis of two key residues proximal to the active site (K146R and Q183A) significantly improved the nuclease activity, suggesting implications for evolutionary relationships with the orthologous nuclease EndA from *S. pneumoniae*.

## 2. Materials and methods   

### 2.1. Cloning, expression and purification of catalytically inactive GBS_NucA (H148A) mutant for crystallization   

The coding sequence for residues Ser42–Asn261 was cloned into the *Not*I/*Bam*HI sites of the pGEXM vector (Moon *et al.*, 2014 ▶). Overexpression of wild-type GBS_NucA in *Escherichia coli* is not possible owing to toxicity, therefore the pGEXM-GBS_NucA construct contained the H148A mutation, which was shown to be catalytically inactive (Derré-Bobillot *et al.*, 2013 ▶). The resulting vector was then transformed into the *E. coli* BL21 Rosetta2 (DE3) cell line for expression. The cells were grown in LB medium at 37°C with shaking at 275 rev min^−1^ to an OD_600 nm_ of 0.7, at which point the temperature was decreased to 18°C for 40 min. Protein expression was induced by the addition of 0.4 m*M* IPTG and was allowed to proceed for 18 h. The cells were lysed by sonication in 25 m*M* Tris pH 7.5, 500 m*M* NaCl and the lysate was cleared by centrifugation. Soluble protein was bound in-batch to glutathione Sepharose 4B resin, and GBS_NucA was cleaved from its GST fusion partner by the addition of TEV protease overnight at 4°C. The resulting supernatant was concentrated and purified by size-exclusion chromatography. Contaminating TEV protease was then removed from the sample by in-batch binding to Ni–NTA resin. GBS_NucA was not retained on the Ni–NTA resin and was concentrated to 27 mg ml^−1^ in a final buffer consisting of 25 m*M* Tris pH 7.5, 100 m*M* NaCl. GBS_NucA behaved as a monodisperse population of monomeric particles in solution, as assayed by size-exclusion chromatography (Supplementary Fig. S1^1^).

### 2.2. Crystallization and structure solution of GBS_NucA   

GBS_NucA apoprotein crystals were grown using the hanging-drop vapor-diffusion technique (Chayen, 1998 ▶) by mixing 2 µl NucA (27 mg ml^−1^) in 25 m*M* Tris pH 7.5, 100 m*M* NaCl with 2 µl reservoir solution consisting of 1.6 *M* ammonium sulfate, 0.1 *M* sodium citrate pH 4.5, 200 m*M* NaCl. For data collection, the crystals were transferred to a cryoprotectant solution consisting of 1.8 *M* ammonium sulfate, 0.1 *M* sodium citrate pH 4.5, 250 m*M* NaCl, 15%(*v*/*v*) ethylene glycol. The crystals were looped and flash-cooled in liquid nitrogen.

The 2.0 Å resolution structure of Mg^2+^-bound GBS_NucA was obtained from a crystallization experiment attempting to produce a protein–DNA complex. The protein:DNA sample consisted of 27 mg ml^−1^ NucA mixed in a 1:1.36 molar ratio of protein to 8-mer duplex DNA (5′-GCGATCGC-3′) in the presence of 16.5 m*M* Tris pH 7.5, 65 m*M* NaCl, 20 m*M* MES pH 6.5 and 2 m*M* MgCl_2_. For crystallization, 300 nl of the protein:DNA sample was mixed with 300 nl reservoir solution (50 m*M* HEPES pH 7.0, 10 m*M* MgCl_2_, 1.6 *M* ammonium sulfate) and the crystals were grown *via* sitting-drop vapor diffusion (Chayen, 1998 ▶). For data collection, 2 µl of a cryoprotectant solution consisting of 50 m*M* HEPES pH 7.0, 100 m*M* NaCl, 10 m*M* MgCl_2_, 1.8 *M* ammonium sulfate, 20%(*v*/*v*) ethylene glycol were slowly added to the crystallization drop. The crystal was looped and flash-cooled in liquid nitrogen.

Data for the GBS_NucA apoprotein 1.5 Å high-resolution data set were collected on the SER-CAT BM-22 beamline at the APS, while all other data sets were collected using an in-house Rigaku MicroMax-007 HF generator equipped with VariMax HF mirrors and a Saturn CCD detector. The data sets for the 1.5 and 2.0 Å resolution structures were refined using *HKL*-2000 (Otwinowski & Minor, 1997 ▶), while *HKL*-3000 (Minor *et al.*, 2006 ▶) was used for the 1.6 Å resolution data. Molecular replacement with *Phaser* (McCoy, 2007 ▶) in the *PHENIX* (Adams *et al.*, 2010 ▶; Terwilliger *et al.*, 2008 ▶) suite was used to solve the phase problem using EndA from *S. pneumoniae* as the search model (PDB entry 3owv; Moon *et al.*, 2011 ▶). A partially refined model of this structure (space group *P*1) was then used as a search model in molecular replacement against the 1.5 Å resolution data set. The same *R*_free_ reflections were used for each of the high-resolution data sets. The 2.0 Å resolution Mg^2+^-bound GBS_NucA data set was solved by molecular replacement (space group *P*6_3_) using the structure from the 1.5 Å resolution apoprotein data set as a search model. The 2.0 Å resolution data set was twinned and was refined with a twin fraction of 0.43 using the twin law (*h*, −*h* − *k*, −*l*) in the refinement (Afonine *et al.*, 2012 ▶). The final models were obtained by iterative cycles of model building in *Coot* (Emsley & Cowtan, 2004 ▶; Emsley *et al.*, 2010 ▶) and refinement in *PHENIX* (Adams *et al.*, 2010 ▶; Terwilliger *et al.*, 2008 ▶). Data-collection and refinement statistics are included in Table 1 ▶.

### 2.3. Generation of GBS_NucA mutants on the H148G background   

The H148G mutant of GBS_NucA was generated using QuikChange mutagenesis. Other GBS_NucA mutants were then generated on the H148G background using the same mutagenesis technique. These mutants were then expressed and purified using the same protocol as for GBS_NucA (H148A), lacking only the in-batch Ni–NTA binding step used to remove residual 6×His-tagged TEV protease. All mutants behaved indistinguishably from GBS_NucA (H148A) in size-exclusion chromatography experiments.

### 2.4. Evaluation of GBS_NucA mutants using imidazole chemical rescue   

GBS_NucA proteins lacking His148 were diluted to 20 n*M* in activity buffer (20 m*M* MES pH 6.5, 0.1 m*M* MgCl_2_) and were incubated with 15 ng µl^−1^ supercoiled pBluescript SK(+) plasmid for 40–45 min in the presence or absence of 30 m*M* imidazole pH 6.5. This concentration of imidazole was chosen for these reactions both to induce maximal nuclease activity and because all phases (nicked open circle, linearized and degraded) of digestion are visible. The reactions were quenched by the addition of loading dye containing EDTA. Samples were run on an 0.8%(*w*/*v*) agarose gel dissolved in 1× Tris–acetate–EDTA buffer. DNA species in the gel were visualized by ethidium bromide staining, scanned with a Typhoon fluorescence imager and analyzed using *ImageQuant TL*. Nuclease activity assays were performed in triplicate.

### 2.5. Single radial enzyme diffusion (SRED) assay   

Agarose [1%(*w*/*v*)] was dissolved in activity buffer (20 m*M* MES pH 6.5, 0.1 m*M* MgCl_2_ with or without 30 m*M* imidazole pH 6.5). High-molecular-weight salmon sperm DNA (30 µg ml^−1^) and ethidium bromide (1 µg ml^−1^) were added immediately prior to casting. GBS_NucA proteins were diluted to 1 mg ml^−1^ in protein storage buffer (25 m*M* Tris pH 7.5, 100 m*M* NaCl) and 1 µl of each protein solution was placed in a small well on the SRED plates. 0.5 µl of DNase I (1 unit µl^−1^) was added to each plate as a positive control. The plates were incubated overnight at 37°C. Nuclease activity is identified by the visualization of a dark ‘halo’ around the well site, as observed under UV_254 nm_ illumination (Nadano *et al.*, 1993 ▶). The relative extent of nuclease activity for each protein was determined by measurement of the radius of the halo and was calculated as a percentage of the H148G background activity. These experiments were performed in triplicate.

## 3. Results   

### 3.1. Crystal structure of NucA from *S. agalactiae*   

**Figure 1:**
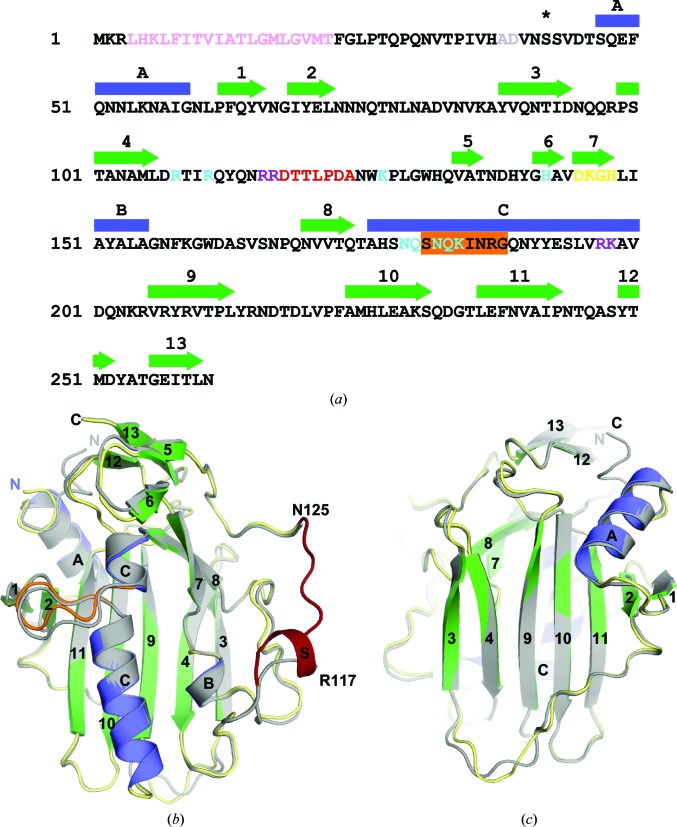
Sequence and structure of NucA. (*a*) Sequence of full-length GBS_NucA. The putative transmembrane secretion signal is shown in pink. Protease cleavage is thought to occur between residues Ala38 and Asp39 (gray). This region is not included in the crystallization construct (starting at Ser42, black asterisk). The D*X*GH motif is colored in yellow. Mutations hypothesized to interfere with DNA binding are marked in purple and all other mutations used in this study are marked in cyan. Residues along the substrate-binding loop are shown in red. ‘Finger loop’ residues are boxed in orange. Secondary-structural elements are shown above the sequence, with α-helices (blue) labeled alphabetically and β-strands (green) numbered. (*b*) Superposition of GBS_NucA apoprotein (gray) and Mg^2+^-bound GBS_NucA (α-helices in blue, β-strands in green and coils in yellow) as viewed from the ‘front’ face of the enzyme. α-Helices and β-strands are colored and numbered as described in (*a*). The ‘finger loop’ insertion in α-helix C is shown in orange. Disordered residues of the substrate-binding loop in the apoprotein are numbered in black and the ordered substrate-binding loop from the Mg^2+^-bound GBS_NucA is shown in red. (*c*) Superposition of GBS_NucA in space group *P*1 (gray) and *P*6_3_ (α-helices in blue, β-strands in green and coils in yellow) as viewed from the ‘back’ face, a 180° vertical rotation from (*b*). Structural figures were created using *PyMOL* (http://www.pymol.org), using molecule *A* from each space group.

The nuclease domain of NucA (Ser42–Asn261), lacking the N-terminal transmembrane secretion signal sequence (Derré-Bobillot *et al.*, 2013 ▶), was bacterially expressed and purified for crystallization (Fig. 1 ▶ *a*). Because wild-type GBS_NucA is difficult to overexpress in *E. coli*, an inactive form of the enzyme, H148A, was used. A 1.5 Å high-resolution X-ray crystal structure of the GBS_NucA (H148A) apoprotein was obtained in space group *P*1 in the absence of a divalent metal (Table 1 ▶). Two molecules of GBS_NucA were present in the asymmetric unit. This structure revealed that the global structure of NucA is comprised of a central antiparallel β-sheet flanked on the ‘front’ face by the ββα metal-finger (ββα-Me) motif that creates the compact active center, leaving the ‘back’ face of the β-sheet largely open to solvent (Figs. 1 ▶ *b* and 1 ▶ *c*). The ββα motif is comprised of β-strands 7 and 8, the latter of which connects directly to α-helix C. The compact active site is outlined by this motif and its deepest recess is formed by the core β-sheet (β-strands 4, 9 and 10). Because the crystallization conditions lacked divalent metal ions, the active site was solvated but otherwise empty. The final model is comprised of nearly the entire nuclease domain, lacking only the coil connecting β-strands 4 and 5 (Fig. 1 ▶ *b*).

**Figure 2:**
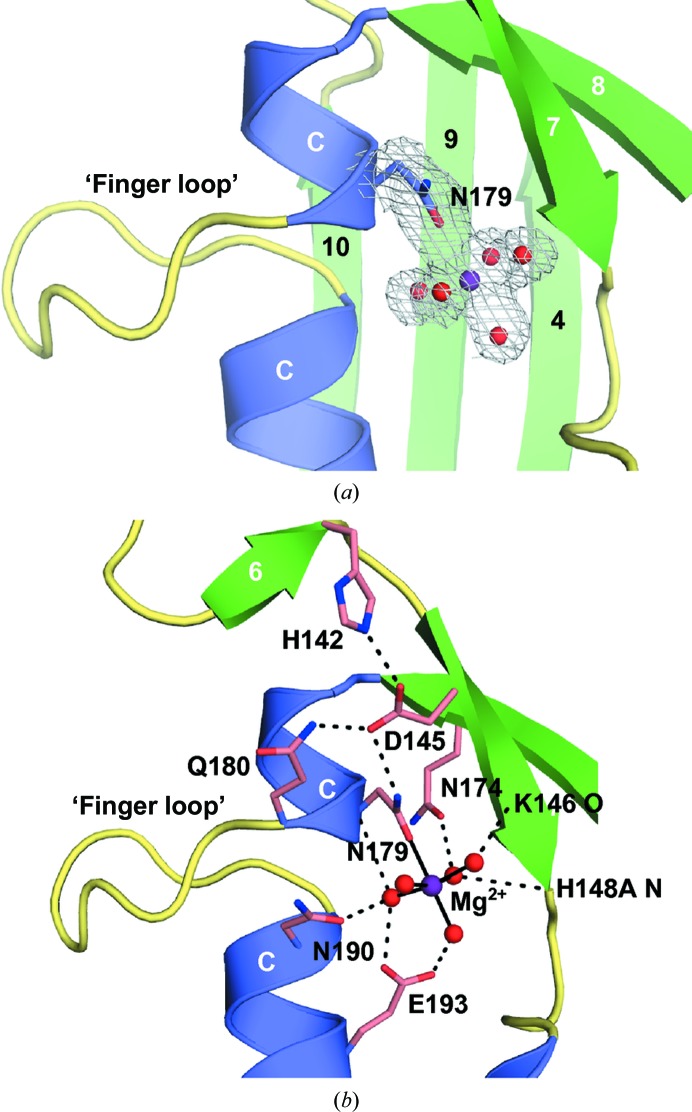
Topology of the NucA active center. (*a*) Ribbon diagram portraying the NucA (H148A) ββα-Me finger motif: α-helices are shown in blue and β-­strands in green. The hydrated Mg^2+^ ion (purple; water molecules in red) is bound within the active site, chelated directly by Asn179 (blue). A 2*F*_o_ − *F*_c_ electron-density map for this cluster (gray) is contoured at 1σ. (*b*) Detailed rendering of interactions near the active center. Chelation of the divalent Mg^2+^ ion (purple) by Asn179 and water molecules (2.1 Å distance) is shown as solid black lines. An extensive hydrogen-bonding network (dashed black lines) connects second-shell and third-shell residues (pink) to the active center.

GBS_NucA was also crystallized in space group *P*6_3_ in the presence of a divalent Mg^2+^ ion and a putative substrate DNA, although there was no visible electron density for the DNA (Table 1 ▶). These crystals diffracted to 2.0 Å resolution and contained four molecules in the asymmetric unit, each with a bound hydrated magnesium ion in the active site (Fig. 2 ▶ *a*, Supplementary Fig. S2 and Supplementary Table S1 ▶). Asn179 is the only residue to directly interact with the metal. All other interactions are mediated through the water molecules that complete the hydration shell to either the backbone (H148A N, Lys146 O and Asn179 O) or to the side chains of residues Asn174, Asn190 and Glu193 (Fig. 2 ▶ *b*). An intricate network of putative hydrogen-bonding interactions links the metal in the active center through Asn179 to the distally located Gln180 and His142, using Asp145 as the intermediary. This network is also present in the apoprotein, where the metal-binding site is unoccupied. The coordination of the hydrated divalent metal and the configuration of residues in the catalytic center of GBS_NucA (Fig. 2 ▶ *b*) are nearly identical to those of DRGH nucleases EndA (*S. pneumoniae*) and NucA (*Anabaena* sp.). Therefore, GBS_NucA is likely to use a similar reaction mechanism (Ghosh *et al.*, 2005 ▶; Moon *et al.*, 2011 ▶).

Comparison of the NucA (H148A) structures in the different space groups reveals that the protein core is nearly identical regardless of packing interactions, although slight variations can be observed in several external loop regions and in the conformation of the N-terminus (r.m.s.d. of 0.285 Å over 159 C^α^ atoms; Figs. 1 ▶ *b* and 1 ▶ *c*). The most significant difference is observed in the ordering of the coil between β-strands 4 and 5 of molecules *A* and *B* in the *P*6_3_ crystal form. This is the first reported structure of a *Streptococcus* sp. nuclease in which this loop is ordered. Residues Arg116–Asp118 along this loop form a short α-helix (henceforth referred to as α-helix S), although there are no such indications of helical propensity in Arg116–Asp118 in the *P*1 crystal form. Given that this coil is entirely disordered in the *P*1 crystal form and is incomplete in molecules *C* and *D* of the *P*6_3_ crystal form, we hypothesize that it is highly mobile in the absence of a DNA substrate. Since residues along this loop in EndA (Arg127/Lys128) have been shown to strongly influence DNA binding (Moon *et al.*, 2011 ▶) and these residues appear to be conserved in GBS_NucA (Arg116/Arg117), this region might also act as a substrate-binding loop in this enzyme.

### 3.2. Structural comparisons of GBS_NucA to other ββα-Me nucleases   

**Figure 3:**
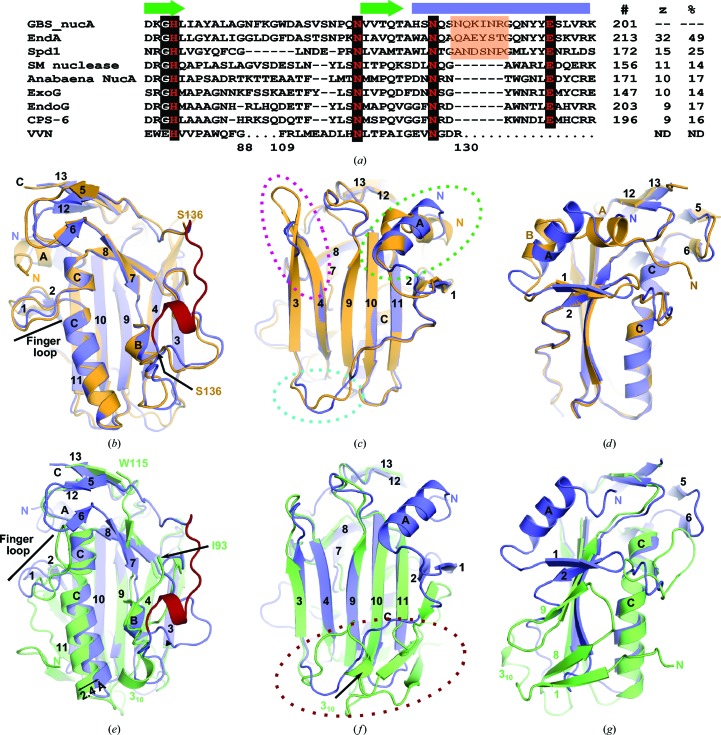
Structural homology with related sequence/structure-nonspecific nucleases. (*a*) Sequence alignment of the ββα-Me finger motif from structurally similar nucleases, as calculated using the *DALI *server (Holm & Rosenström, 2010 ▶). Nucleases included in this list include EndA from *S. pneumoniae*, Spd1 from *S. pyogenes* encoded by the SF370.1 prophage, SM nuclease from *Serratia marcescens* and NucA from *Anabaena* sp. The positions of the β-strands are marked by green arrows and the α-helix by a blue rectangle (corresponding to β-strands 7–8 and α-helix C in GBS_NucA). Conserved residues are boxed in black. Key residues for catalytic function are shown in red. The ‘finger loop’ from *Streptococcus* sp. nucleases is boxed in orange. Residue numbers are indicated to the right of the alignment, and the *Z*-scores and structure-based sequence identities (as calculated by the *DALI* server) are listed on the far right. Sequence interruptions of the VVN nuclease sequence are shown as repeated dots, with the residue numbers below. (*b*, *c*, *d*) Structural superposition of GBS_NucA (H148A) (blue) and EndA (H160A) (PDB entry 3owv; Moon *et al.*, 2011 ▶; orange), emphasizing the ‘front’ (*b*), ‘back’ (*c*) and ‘side’ (*d*) faces of the enzymes. (*e*, *f*, *g*) Structural superposition of GBS_NucA (H148A) (blue) and Spd1 (PDB entry 2xgr (Korczynska *et al.*, 2012 ▶; green), emphasizing the ‘front’ (*e*), ‘back’ (*f*) and ‘side’ (*g*) faces of the enzymes. Dashed circles highlight regions of structural dissimilarity.

A structural homology search using the *DALI* server (http://ekhidna.biocenter.helsinki.fi/dali_server/start; Holm & Rosenström, 2010 ▶) indicated that GBS_NucA shares the highest degree of structural similarity with EndA from *S. pneumoniae* (*Z*-score 32.5) and Spd1 from *S. pyogenes* (*Z*-score 15.2) (Fig. 3 ▶). The *DALI* server calculated the structure-based sequence identity of NucA to be 49 and 25%, respectively (Fig. 3 ▶ *a*). NucA appears to be more distantly similar to other classical DRGH nucleases such as SM nuclease from *Serratia marcescens*, NucA from *Anabaena* sp., human mitochondrial ExoG and two orthologs of EndoG from *Drosophila melanogaster* and *Caenorhabditis elegans* (CPS-6). These more distantly related nucleases had *Z*-scores of less than 12 and nearly random structurally homologous sequence identities. Despite the low overall sequence and structural conservation, the ββα motifs of these nucleases aligned well, as did the histidine residue thought to serve as the general base and the asparagine chelating the divalent metal in the active site (Fig. 3 ▶ *a*)

**Figure 4:**
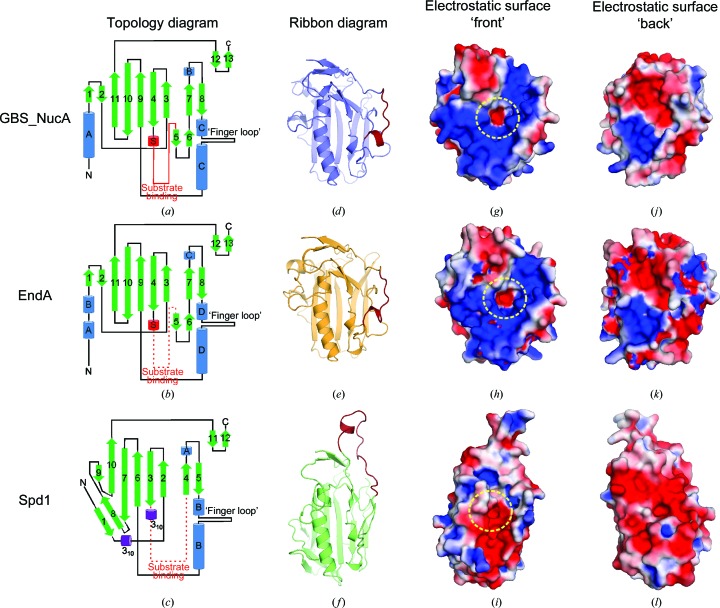
Comparison of topology and electrostatic properties. (*a*, *b*, *c*) Topology and connectivity diagrams for GBS_NucA (H148A) (*a*), EndA (H160A) (*b*) and Spd1 (*c*). (*d*, *e*, *f*) Ribbon diagrams, viewing the ‘front’ face of the enzymes, with the substrate-binding loops shown in red. For EndA (H160A) (*e*), this region was modeled on that of NucA (H148A) in space group *P*6_3_ and the correct EndA sequence was threaded onto this structure. For Spd1 (*f*), this region lacked significant structural homology to either NucA or EndA. Therefore, the conformation of this loop was generated *de novo* using *SWISS-MODEL* (for details, see Supporting Information; Arnold *et al.*, 2006 ▶). (*g*–*l*) Electrostatic surface potentials for NucA (H148A), EndA (H160A) and Spd1. For each model, any incomplete side chains were rebuilt using preferred side-chain rotamers (Lovell *et al.*, 2000 ▶) and the least favored of all alternate conformations were removed. Electrostatic surface potentials were calculated using the *Adaptive Poisson-Boltzmann Solver* tool in *PyMOL* (Baker *et al.*, 2001 ▶) and range from −2*kT* e^−1^ (electronegative, red) to 22*kT* e^−1^ (electropositive, blue). Regions of neutral charge are shown in white. The location of the active site is highlighted by a dashed yellow circle.

Structural superposition of GBS_NucA (H148A) with the most closely related EndA (PDB entry 3owv; Moon *et al.*, 2010 ▶) reveals that the two nucleases have a nearly identical structure (r.m.s.d. of 1.1 Å over 192 C^α^ atoms; Figs. 3 ▶ *b*, 3 ▶ *c* and 3 ▶ *d*) and electrostatic surface potential (Figs. 4 ▶ *g*, 4 ▶ *h*, 4 ▶ *j* and 4*k*). The most notable difference is in the conformation of the N-termini: in GBS_NucA the N-terminus is comprised of a short coil that is somewhat variable in structure, leading to an 11-residue α-helix (αA, Gln48–Ile58). The N-terminus of EndA, by comparison, has a longer coil which begins on the ‘front’ face of the enzyme and travels across a hydrophobic ‘shoulder’ of the enzyme to the ‘back’ face (Fig. 3 ▶ *d*). There it forms two smaller α-helices (αA, Gln51–Val57; αB, Asp60–Gln65) residing in a position similar to αA in GBS_NucA (Figs. 4 ▶ *a* and 4 ▶ *b*). Although the reasons for this structural difference are unclear, the variation could be related to the localization of each enzyme: NucA is secreted into the extracellular medium, while EndA can either be secreted or membrane-associated as a component of the competence complex (Lacks & Neuberger, 1975 ▶; Puyet *et al.*, 1990 ▶). Other small structural variations can be observed in external loop regions, which might be influenced by differences in both sequence and crystal-packing interactions (Fig. 3 ▶ *c*).

GBS_NucA aligns less well with the structure of Spd1 (r.m.s.d. of 1.9 Å over 141 C^α^ atoms using PDB entry 2xgr; Korczynska *et al.*, 2012 ▶; Figs. 3 ▶ *e*, 3 ▶ *f* and 3 ▶ *g*). The central β-sheet is largely conserved (Figs. 3 ▶ *e* and 3 ▶ *f*), but there is an alteration in various components of the ββα motif (Fig. 3 ▶ *e*). The putative substrate-binding loop in Spd1 is considerably longer than that of NucA, and its C-terminal end slightly alters the trajectory of the first of the two β-strands (β4) of the ββα motif. The coil connecting β-strands 7 and 8 in NucA is longer than in Spd1: its structure deviates after the short α-helix (αB in NucA and αA in Spd1; Fig. 4 ▶ *c*) until it rejoins the following β-strand. Additionally, the long α-helix of the ββα motif in Spd1 (αB) aligns well with the N-terminal portion of the helix in NucA (αD), but the C-terminal end of the helix, below the ‘finger loop’, deviates by 2.4 Å. These changes drastically change the disposition of charges on the electrostatic surface of the ‘front’ face of Spd1, giving this enzyme a surprisingly dense arrangement of negatively charged residues in the region that is most likely to bind DNA (Figs. 4 ▶ *i* and 4 ▶ *l*).

The most prominent difference between the structures of NucA and Spd1 is the topology of the N-terminus. Spd1 does not contain an N-terminal α-helix on the ‘back’ face of the enzyme. Rather, the Spd1 N-terminus is comprised of a coil originating on the ‘front’ face, relatively near αB. As this coil travels from the ‘front’ face to the ‘back’, it forms a short β-strand (β1; Figs. 3 ▶ *f*, 3 ▶ *g* and 4 ▶ *c*), continuing on across the ‘back’ face to β-strand 2 at the opposite end of the central β-sheet. Putative hydrogen-bonding interactions exist between β-strands 1 and 8, forming a two-stranded antiparallel β-sheet that is not present in GBS_NucA or EndA. The placement of β-strand 8 is intriguing, since its N-terminal end also makes as many as four possible hydrogen bonds with β-strand 7. This interaction alters the contour of the central β-sheet, with β-strand 8 forming a ‘wedge’ to open the sheet between β-strands 7 and 10 (Figs. 3 ▶ *f* and 4 ▶ *c*).

One of the most intriguing aspects of the GBS_NucA, EndA and Spd1 structures is the structural conservation of the ‘finger loop’ bisecting the long α-helix bordering the active site (Figs. 2 ▶ *a* and 3 ▶ *a*). This extrahelical extrusion from the long α-helix component of the ββα motif has very similar structures in GBS_NucA and EndA (Fig. 3 ▶ *b*), but displays a strikingly different structure in Spd1 (Fig. 3 ▶ *e*). It should be noted that the ‘finger loop’ is currently only found in structure/sequence-nonspecific DNA/RNA nucleases from bacteria in the genus *Streptococcus*. Even among these three enzymes, although the length of the ‘finger loop’ is conserved, the sequence is not (Fig. 3 ▶ *a*). There does appear to be a glycine residue at the C-terminal end of the extrusion (Gly188, Gly200 and Gly159 in NucA, EndA and Spd1, respectively) where the loop rejoins the α-helix.

### 3.3. Biochemical characterization of NucA surface residues: effects on substrate binding and catalysis   

**Figure 5:**
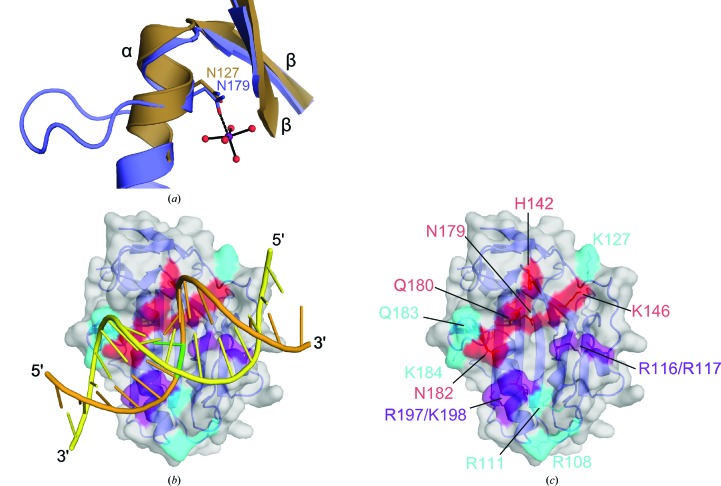
Identification of NucA surface residues for mutagenesis and biochemical characterization. (*a*) Least-squares superposition (LSQ) of the ββα motifs from GBS_NucA (H148A) (Val144–Gly147, Val171–Thr175 and Ala176–Asn179, blue) and VVN nuclease (PDB entry 2ivk; Wang *et al.*, 2007 ▶; Ile76–Glu79, Leu119–Ile123 and Gly124–Asn127, brown). (*b*) Modeling of the 16 bp duplex DNA substrate (orange) from VVN nuclease onto the structure of GBS_NucA (H148A) (blue, molecular surface in gray) based on the LSQ superposition of their ββα motifs. The scissile DNA strand is shown in orange, with the location of the scissile phosphate highlighted in green. The complementary DNA strand is shown in yellow. (*c*) GBS_NucA (H148A) surface residues chosen for mutagenesis with likelihood of involvement in catalysis (red) or DNA substrate binding (cyan or purple).

Thus far, there are no published structures of EndA, Spd1 or other similar ββα metal-finger nucleases in complex with a bound DNA or RNA substrate. Similar attempts to co-crystallize such a complex with GBS_NucA have likewise failed (unpublished data; see Supporting Information). However, X-ray crystal structures exist for the ββα-Me periplasmic nuclease VVN from *Vibrio vulnificus* in complex with duplex DNA [PDB entries 1oup (Li *et al.*, 2003 ▶) and 2ivk (Wang *et al.*, 2007 ▶)]. VVN is unreleated to the *Streptococcus* sp. ββα-Me nucleases and exhibits no sequence conservation. Alhough the *DALI* server was unable to identify any discernable structural homology with VVN, superposition of the ββα motif alone (Glu77–Ala80, Thr120–Ile123 and Gly124–Asn127 in VVN) yields admirable results (Fig. 5 ▶ *a*). Using this alignment, the DNA substrates bound to VVN were docked onto the surface of NucA (Fig. 5 ▶ *b*) and lie along a shallow cleft in close contact with the active site. The ‘front’ face of NucA was then surveyed for surface-accessible residues proximal to the active site which might have the capacity to influence catalysis (Fig. 5 ▶ *c*, red) or to interact with the DNA substrate (Fig. 5 ▶ *c*, cyan or purple). These residues were subjected to alanine-substitution mutagenesis and were subsequently assayed for nuclease activity.

Because overexpression of wild-type GBS_NucA in *E. coli* is difficult owing to toxicity (Derré-Bobillot *et al.*, 2013 ▶), all biochemical analyses of surface mutations was performed on the background of a catalytically deactivated mutant, the activity of which could be re-introduced using an imidazole chemical rescue technique (Midon *et al.*, 2012 ▶; Moon *et al.*, 2011 ▶). This technique is founded on the basic principle that exogenously added imidazole functions as an effective mimic of the absent histidine thought to serve as the general base (Lehoux & Mitra, 1999 ▶). Like the histidine, imidazole could abstract a proton from a nearby water molecule, activating it for nucleophilic attack on the scissile phosphate (Ghosh *et al.*, 2005 ▶).

**Figure 6:**
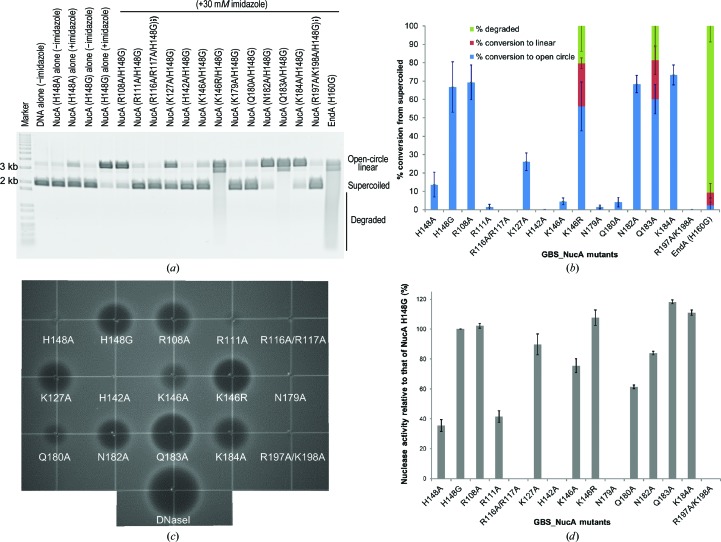
Imidazole chemical rescue assays for nuclease activity in GBS_NucA mutants. (*a*) Nuclease-activity assays for GBS_NucA mutants on the background of H148G. The DNA substrate used for this assay is a supercoiled plasmid, which is then nicked to the open-circle form. The open-circle form accumulates double-strand breaks, linearizing the plasmid, and is further degraded to lower molecular-weight fragments. (*b*) The percentage of each form was calculated using *ImageQuant TL* (GE Healthcare) and the standard deviation for each calculation is indicated by the error bars. All reactions were performed in triplicate. (*c*) Single radial enzyme diffusion (SRED) assay for GBS_NucA mutants performed in the presence of 30 m*M* imidazole. The extent of nuclease degradation is indicated by the size of the ‘halo’ and results from a fluorescence decrease correlated to a decrease in double-stranded DNA substrate. (*d*) Nuclease activity for each GBS_NucA mutant was calculated by measuring the ‘halo’ radius, normalizing to the amount of activity of the H148G mutant (calculated as 100%). All reactions were performed in triplicate, with the error bars indicating the standard deviation for each calculation.

In this study, the inactive GBS_NucA (H148A) mutant was used for crystallization. However, a glycine substitution at the equivalent position in EndA enhanced imidazole rescue, presumably owing to decreased steric hindrance between the C^β^ atom and the imidazole ring (Moon *et al.*, 2011 ▶). Therefore, the activity of the H148G mutant was also assayed in the plasmid conversion assay. Because GBS_NucA (H148A) has very little activity in the imidazole chemical rescue assay and glycine substitution at this position provides greatly enhanced substrate degradation under the same conditions (Fig. 6 ▶), all of the surface mutations were generated on the background of the H148G mutant.

It should be noted that the conditions for the imidazole rescue assay were optimized to produce maximal GBS_NucA (H148G) activity, and that the final conditions differ drastically from those used for EndA (H160G) (Midon *et al.*, 2012 ▶; Moon *et al.*, 2011 ▶). The pH optimum for GBS_NucA is considerably lower than for EndA (pH 6.5 *versus* pH 8, respectively) and the divalent-ion concentration is two orders of magnitude lower (0.1 m*M* versus 10 m*M* for EndA). Additionally, EndA (H160G) appears to be a more active enzyme than NucA (H148G), given that substantially more of the plasmid substrate is degraded by EndA (H160G) to lower molecular-weight fragments in the same amount of time (Fig. 6 ▶ *a*).

Of the mutants generated on the NucA (H148G) background, three (R108A/H148G, N182A/H148G and K184A/H148G) did not differ significantly from the H148G mutant alone either in the plasmid conversion assay or in the SRED assay (Fig. 6 ▶). Conversely, the N179A/H148G mutant had nearly undetectable levels of activity in each assay. Since Asn179 is the only protein side chain directly chelating the hydrated divalent Mg^2+^ ion in the active site, these results provide confirmation that the plasmid conversion and SRED assays are performing as expected. The H142A/H148G mutant and the double mutants R116A/R117A/H148G and R197A/K198G/H148G also exhibited no detectable activity. Surprisingly, the Q183A/H148G and K146R/H148G mutants exhibited a significantly increased activity relative to the H148G mutation alone.

The nuclease-activity analysis yielded intriguingly different results for four mutants in the plasmid conversion assay *versus* the SRED assay. Of these, R111A/H148G, K146A/H148G and Q180A/H148G all displayed severely diminished activity in the plasmid conversion assay (Figs. 6 ▶ *a* and 6 ▶ *b*) but showed substantially more activity in the SRED assay (41.5 ± 3.8%, 75.6 ± 4.6% and 61.4 ± 1.2% of that of H148G alone, respectively; Figs. 6 ▶ *c* and 6 ▶ *d*). For the K127A/H148G mutant, the difference between the assays was less profound, since the K127A/H148G mutant converted 26.2 ± 4.8% of the supercoiled plasmid substrate to the open-circle form (*versus* 66.8 ± 13.7% for the H148G mutation alone). In the SRED assay, the K127A/H148G mutant formed a substrate-degradation ‘halo’ that approached the size of the ‘halo’ made by the H148G mutation alone (89.7 ± 7% of that for the H148G mutant).

## 4. Discussion   

Here, we present X-ray crystal structures and biochemical characterization of the DNA/RNA structure/sequence-nonspecific nuclease NucA from *S. agalactiae*.

### 4.1. Sequence conservation among the D*X*GH nucleases   

**Figure 7:**
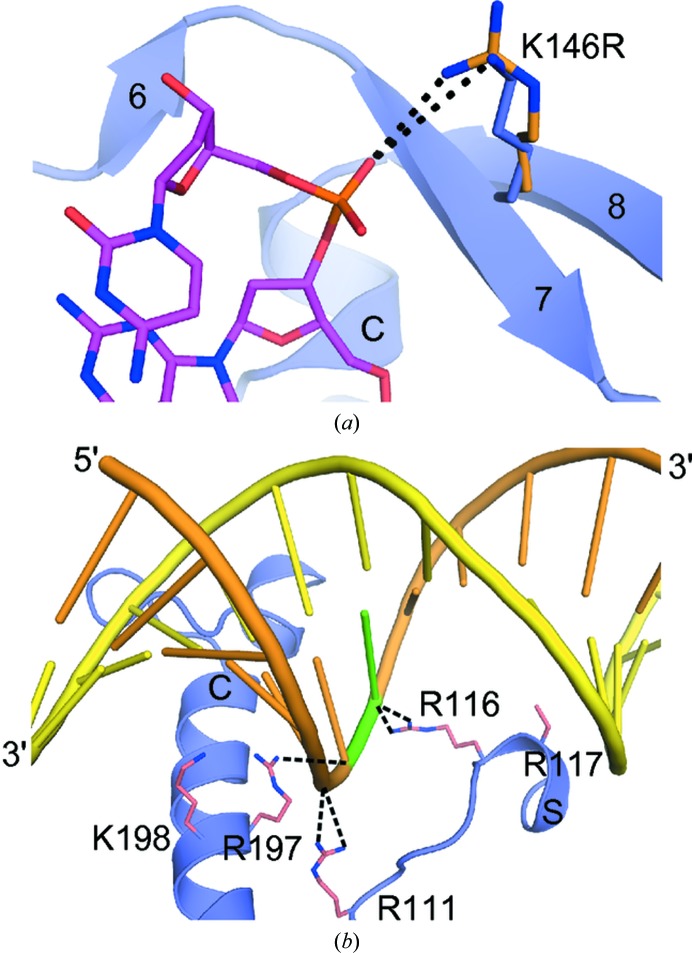
Structural and biochemical implications for DNA substrate binding and catalysis. (*a*) Potential interactions of Lys146 in GBS_NucA (blue) with the 8-mer duplex DNA substrate from VVN nuclease (purple, PDB entry 1oup; Li *et al.*, 2003 ▶) compared with an arginine side chain (orange) at this position. (*b*) Specific GBS_NucA residues appear to be critical for DNA substrate binding. The 16-mer uncleaved duplex DNA substrate (scissile strand in orange, complementary strand in yellow) from VVN nuclease was modeled bound to GBS_NucA (blue ribbon). Side chains from GBS_NucA are shown in pink, with putative hydrogen-bonding interactions with the DNA substrate shown as black dashed lines.

Although the DRGH component of ββα-Me nucleases is thought to be the canonical active-site motif [in EndA (Midon *et al.*, 2011 ▶), *Anabaena* sp. NucA (Ghosh *et al.*, 2005 ▶), SM nuclease (Shlyapnikov *et al.*, 2000 ▶) and EndoG (Loll *et al.*, 2009 ▶), for example], several nucleases with variant sequences have recently been identified. Spd1, Sda1 and GBS_NucA from *S. agalactiae* each have variations in this motif: NRGH for Spd1 (Korczynska *et al.*, 2012 ▶) and DRSH for Sda1 (Aziz *et al.*, 2004 ▶). YbfB from *Lactobacillus lactis* has been identified as a candidate nuclease but contains an ARGH motif. This variation in YbfB is intriguing, since similar alanine substitutions in EndA (Moon *et al.*, 2010 ▶) and *Anabaena* sp. NucA (Ghosh *et al.*, 2005 ▶) were severely detrimental to catalytic activity and could contribute to the DNase-negative phenotype of *L. lactis* (Le Loir *et al.*, 1998 ▶). NucA from *S. agalactiae* has a DKGH sequence, which is a conservative difference that could possibly be explained as a polymorphism between *Streptococcus* species. Intriguingly, conversion of this lysine in NucA from its DKGH motif to the canonical DRGH sequence generated a significantly more active nuclease (Fig. 6 ▶). Structurally, the increased length of the arginine side chain at this position could bring the positively charged guanido group into closer proximity to a DNA substrate, allowing tighter binding (Fig. 7 ▶ *a*).

### 4.2. Implications for DNA binding, substrate selection and catalysis   

Since soaking of apoprotein crystals and co-crystallization with DNA substrates of varying lengths, structures and sequences yielded no visible electron density for a bound oligonucleotide (data not shown; see Supporting Information), we used site-directed mutagenesis along the putative substrate-binding cleft to examine the roles that individual amino acids may play in substrate binding. Of the residues mutated in this study, Arg111, Lys146, Arg116/Arg117 and Arg197/Lys198 are the most likely to electrostatically influence DNA/RNA substrate binding. Arg108 lies farthest from the catalytic center and is therefore unlikely to play a direct role in catalysis (Fig. 5 ▶ *c*). Interestingly, Arg111, Arg116 and Arg197 all lie within hydrogen-bonding distance of the modelled scissile DNA strand (Fig. 7 ▶ *b*). For efficient binding and cleavage, this DNA strand would likely lie in closest proximity to the surface of the enzyme. In duplex DNA substrates, the complementary strand makes fewer contacts with the protein surface. That the majority of contacts occur with the scissile DNA strand correlates well with the ability of GBS_NucA to bind and cleave both double-stranded and single-stranded DNA substrates (Derré-Bobillot *et al.*, 2013 ▶).

Previous studies with the related EndA nuclease from *S. pneumoniae* yielded some information on DNA binding and catalysis that is likely to hold true for GBS_NucA as well. Double mutation of EndA Arg128/Lys129 and Arg209/Lys210 (equivalent to Arg116/Arg117 and Arg197/Lys198) to alanine showed a similarly devastating effect on catalysis by EndA (Moon *et al.*, 2011 ▶). DNA-binding studies showed that these mutants were no longer capable of binding the duplex DNA substrate. Analysis of the individual residues showed that in each case alanine substitution of a single residue of each pair slightly decreased DNA binding. However, the resulting mutants showed a greater extent of catalytic impairment for Arg128 and Arg209 compared with the relative decrease in substrate binding. Residues of each pair worked synergistically to bind the DNA substrate, but once bound the first residue of each pair also played a role in catalysis. Similar results were obtained for individual mutants of GBS_NucA. Arg116 and Arg197 were almost solely responsible for the loss of the catalytic activity exhibited by the Arg116/Arg117 and Arg197/Lys198 double mutants (Supplementary Fig. S3). These results are consistent with the structural model of DNA binding to GBS_NucA in that the first of each pair lies in closer proximity to the DNA substrate (Fig. 7 ▶ *b*).

This crystal structure of GBS_NucA is the first of the *Streptococcus* species nucleases to exhibit an ordered substrate-binding loop. Based on the model of DNA bound to GBS_NucA, α-helix S in the substrate-binding loop lies in the major groove of the DNA duplex and contains arginine residues 116 and 117 (Fig. 7 ▶ *b*). Positioning of an electropositive residue in this loop, in close proximity to the scissile phosphate, appears to be required for catalytic activity in nearly all known D*X*GH nucleases, possibly in order to alleviate the charge buildup on this phosphate (Moon *et al.*, 2011 ▶). For GBS_NucA, the structurally equivalent residue would be Arg116 (Fig. 7 ▶ *b*). The substrate-binding loop adopts different structures in Spd1 and *Anabaena* NucA, but Arg90 and Arg93, respectively, have been hypothesized to play a similar role in these enzymes (Korczynska *et al.*, 2012 ▶; Ghosh *et al.*, 2005 ▶). There is no directly structurally homologous residue in the substrate-binding loop in SM nuclease from *Serratia marcescens*, but the guanido group from Arg57, farther along the loop, lies within hydrogen-bonding distance of the scissile phosphate (Shlyapnikov *et al.*, 2000 ▶). Even VVN, which has no detectable structural similarity to the DRGH nucleases beyond the ββα motif, has a positively charged arginine side chain (Arg99) stabilizing the position of this phosphate in the cleaved product complex. Since Arg99 makes a putative hydrogen bond to the VVN post-catalytic product, rather than in the pre-catalytic complex, it has been hypothesized that this interaction preferably stabilizes the product state rather than the transition state, and possibly inhibits the reaction from running in reverse (Li *et al.*, 2003 ▶).

### 4.3. Putative role of the ‘finger loop’ helical extrusion   

Although the ‘finger loops’ in GBS_NucA, EndA and Spd1 exhibit no sequence conservation, the lengths of the extrusions are conserved (Fig. 3 ▶ *a*). Despite the apparent lack of sequence conservation, the ‘finger loops’ of GBS_NucA and EndA are globally similar (Fig. 3 ▶ *b*). The same loop in Spd1, however, displays a very different conformation (Fig. 3 ▶ *e*). In Spd1 this loop lies in close proximity to the proposed DNA-binding cleft and could affect its dimensions and/or substrate-binding affinity. Gln183 on the ‘finger loop’ helical extrusion from α-helix C in GBS_NucA occupies a position very close to the minor groove of the substrate-bound complex and could provide a steric clash with a bound DNA substrate. Therefore, replacement of this glutamine with a smaller alanine side chain could alleviate the steric clash, widen the substrate-binding cleft and allow a duplex substrate to bind more easily. Such a hypothesis is consistent with the observation that the Q183A mutation yielded an enzyme with increased catalytic activity compared with the H148G background (Fig. 6 ▶). That mutagenesis along the ‘finger loop’ affects catalysis is a distinct difference between GBS_NucA and the related EndA nuclease. For EndA, altering the ‘finger loop’ by either mutagenesis or deletion had no effect on DNA binding or catalysis (Moon *et al.*, 2011 ▶).

### 4.4. Comparison of nuclease activity in the plasmid conversion and SRED assays   

Four of the mutants assayed exhibited different extents of imidazole-rescued nuclease activity in the plasmid conversion and SRED assays (Figs. 6 ▶ *b* and 6 ▶ *d*). Such disparities in these assays were not observed for the related DRGH nuclease EndA (Moon *et al.*, 2011 ▶). We currently have no concrete explanation for these differences. All of the mutants (R111A/H148G, K127A/H148G, K146A/H148G and Q180A/H148G) displayed lower catalytic activity in the plasmid conversion assay as opposed to the SRED assay. None of the mutants displayed lower activity in the SRED assay. There are a few possible explanations for this observation, the first of which is that the SRED assay is performed using a considerably higher DNA concentration (30 µg ml^−1^ in the SRED assay *versus* 20 ng total in the plasmid conversion assay) and runs over a longer time scale (17–18 h) compared with the plasmid conversion assay (40 min). Additionally, the plasmid conversion assay is carried out in solution, while the DNA in the SRED assay is somewhat immobilized in the agarose matrix. Therefore, it is possible that mutations decreasing either the substrate-binding affinity or reaction rate may have a greater chance of cleaving an immobilized substrate over a longer reaction time.

## 5. Biological implications   

The GBS_NucA crystal structures allow detailed comparison with related nucleases from other *Streptococcus* species and provides the foundation for structure-driven mutagenesis and biochemical characterization. Such an array of structural information may facilitate rational drug design to inhibit these nucleases in the context of invasive *Streptococcus* sp. infections. Since these nucleases commonly serve as secreted virulence factors for these bacteria, they represent a unique opportunity for drug therapies that specifically target the virulence factors independently of the invading bacteria. This therapeutic strategy could decrease the impact of the infection, while simultaneously minimizing the impact on the natural bacterial flora and fauna of the host.

In recent years, an imbalance in NET production and clearance has been found to play a role in many different types of human diseases: cystic fibrosis, systemic lupus erythematosus and allergic asthma (Cheng & Palaniyar, 2013 ▶; Yu & Su, 2013 ▶). NET-degrading enzymes such as GBS_NucA and EndA may therefore be valuable therapeutic tools for treating such human diseases. Similar therapies are currently in use: deoxyribonuclease-1, for example, is part of a multi-drug therapy to decrease mucosal secretion viscosity in cystic fibrosis (Papayannopoulos *et al.*, 2011 ▶).

## 6. Related literature   

The following reference is cited in the Supporting Information for this article: Laskowski *et al.* (2003 ▶).

## Supplementary Material

PDB reference: GBS_NucA, 4qgo

PDB reference: with bound active-site magnesium, 4qh0

Supporting Information.. DOI: 10.1107/S1399004714019725/mn5067sup1.pdf

## Figures and Tables

**Table 1:** Data-collection and refinement statistics Values in parentheses are for the highest resolution shell. A single crystal was used for data collection in each case.

	NucA (H148A) apo†	NucA (H148A) apo	NucA (H148A) + Mg^2+^
PDB entry		4qgo	4qh0
Data collection			
Space group	*P*1	*P*1	*P*6_3_
Unit-cell parameters			
*a* ()	37.69	37.59	123.72
*b* ()	57.02	57.13	123.72
*c* ()	62.37	62.42	157.40
()	70.56	109.40	90
()	82.89	97.08	90
()	90.17	90.16	120
Resolution range ()	501.6	501.5	502.0
Reflections (measured/unique)	247628/61631	291203/74966	395220/89816
Completeness (%)	95.8 (74.4)	91.4 (96.3)	97.2 (79.9)
Multiplicity	4.0 (1.4)	3.4 (3.9)	4.4 (1.7)
*R*_cryst_	0.098 (0.36)	0.058 (0.26)	0.080 (0.33)
Mean *I*/(*I*)	20.29 (2.14)	27.5 (4.31)	32.67 (2.8)
Monomers per asymmetric unit	2	2	4
Refinement			
Resolution range ()		30.561.50	252.00
*R*_work_/*R*_free_ ‡ (%)		15.69/18.28	15.53/19.05
Unique reflections (working/test)		70302/4656	87733/1976
Water molecules		658	773
Total No. of atoms		4295	7782
Average *B* factor (^2^)			
Protein		11.43	29.84
Ions		29.94	44.05
Water		25.54	36.56
R.m.s.d., bond lengths ()		0.009	0.004
R.m.s.d., bond angles ()		1.237	0.816
Ramachandran plot statistics, residues in			
Allowed regions (%)		100	100

† The lower resolution 1.6 NucA (H148A) data set was used as a search model for molecular replacement for the higher resolution 1.5 data set. The model for the 1.6 data set was not fully refined; therefore, only the statistics for data collection are included.

‡ *R*_work_ = 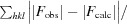 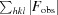, where *R*_free_ is calculated for a randomly chosen 5% of reflections which were not used for structure refinement and *R*_work_ is calculated for the remaining reflections.

## Footnotes

1 Supporting information has been deposited in the IUCr electronic archive (Reference: MN5067).
