# Supplementary material for: Structural characterization of the virulence factor nuclease A from Streptococcus agalactiae
Source: Acta Crystallogr D Biol Crystallogr. 2014 Oct 23;70(Pt 11):2937–49. doi: 10.1107/S1399004714019725 (PMC4220975; doi:10.1107/S1399004714019725)
Supplement: Supplementary file 1 [file d-70-02937-sup1.pdf]

Acta Crystallographica Section D

Volume 70 (2014)

Supporting information for article:

Structural characterization of the virulence factor Nuclease A from *Streptococcus agalactiae*

Andrea F. Moon, Philippe Gaudu and Lars C. Pedersen

S1. Co-crystallization of GBS_NucA/DNA complex

Attempts were made to co-crystallize GBS_NucA with a variety of DNA substrates, including an 11-mer duplex (5'-CGGCCGTACTG-3' and 5'-CAGTACGGCCG-3'), a palindromic 8-mer duplex (5'-GCGATCGC-3'), a short 3'-overhang (5'-CCGCC-3' and 5'-GGCGGTT-3'), a 9-mer nicked single-stranded break duplex, (5'-CGGCGTACG-3', 5'-CGTAC-3', and 5'-pGCCG-3'), and a single-stranded oligonucleotide (5'-CGGCGTACG-3'). All DNA substrates were mixed in equimolar ratios in the presence of 353 mM MES pH 6.5 and 1.75 mM $MgCl_2$, and annealed by denaturation at 94 °C and slow-cooling to 4 °C in a thermalcycler. The annealed DNA was then mixed with GBS_NucA (27 mg ml^{-1}) at a molar ratio of 1-2:1 (DNA:protein) and incubated on ice at 4 °C for 1 hour. The resulting GBS_NucA/DNA complex was then centrifuged at 13000 x g for 2 minutes at 4 °C to pellet any precipitate, and set up in sitting-drop vapor diffusion experiments against commercially-available sparse-matrix crystallization screens. Crystals were obtained from these conditions as specified in Section 2.2 of the main text, but no electron density was visible for a bound DNA substrate.

S2. Comparison of GBS_NucA structure, between space groups P1 and $P6_3$

In space group P1, there were two molecules per asymmetric unit, which superimposed well (RMSD of 0.233 Å over 214 Cα atoms) and displayed only slight alterations of the loops between β-strands 3 and 4 and β-strands 9 and 10 (Suppl. Fig. 1*a*). These alterations are likely caused by crystal packing interactions. Superpositions of the four molecules per asymmetric unit in space group $P6_3$ yielded RMSD values of 0.185-0.445 Å over 210-218 Cα atoms (Suppl. Table 1). Small differences are apparent in the conformation of the N-termini of each molecule (Suppl. Fig. 1*b*), caused by packing interactions and the presence of a three alanine linker that is

a cloning artifact. Additionally, the coil between β-strands 2 and 3 that traverses the ‘back’ face of the enzyme displays two different conformations—the coils in molecules A and B are similar, but distinct from those in molecules C and D (Suppl. Fig. 1*c*).

S3. Generation of Spd1 substrate binding loop using SWISS-MODEL

The crystal structure of Spd1 from *Streptococcus pyogenes* (PDB ID code 2XGR (Korczynska *et al.*, 2012)) was used as the starting model. The least favored of all side chain alternate conformations were deleted, retaining only the highest occupancy conformation (conformation A, where the occupancies were equal). All side chains with missing atoms were manually rebuilt, using the most likely conformation from the Richardson rotamer library with no likely clashes with neighboring residues (Lovell *et al.*, 2003). Using the resulting model, a hypothetical structure for the disordered substrate binding loop was then generated by SWISS-MODEL (Arnold *et al.*, 2006).

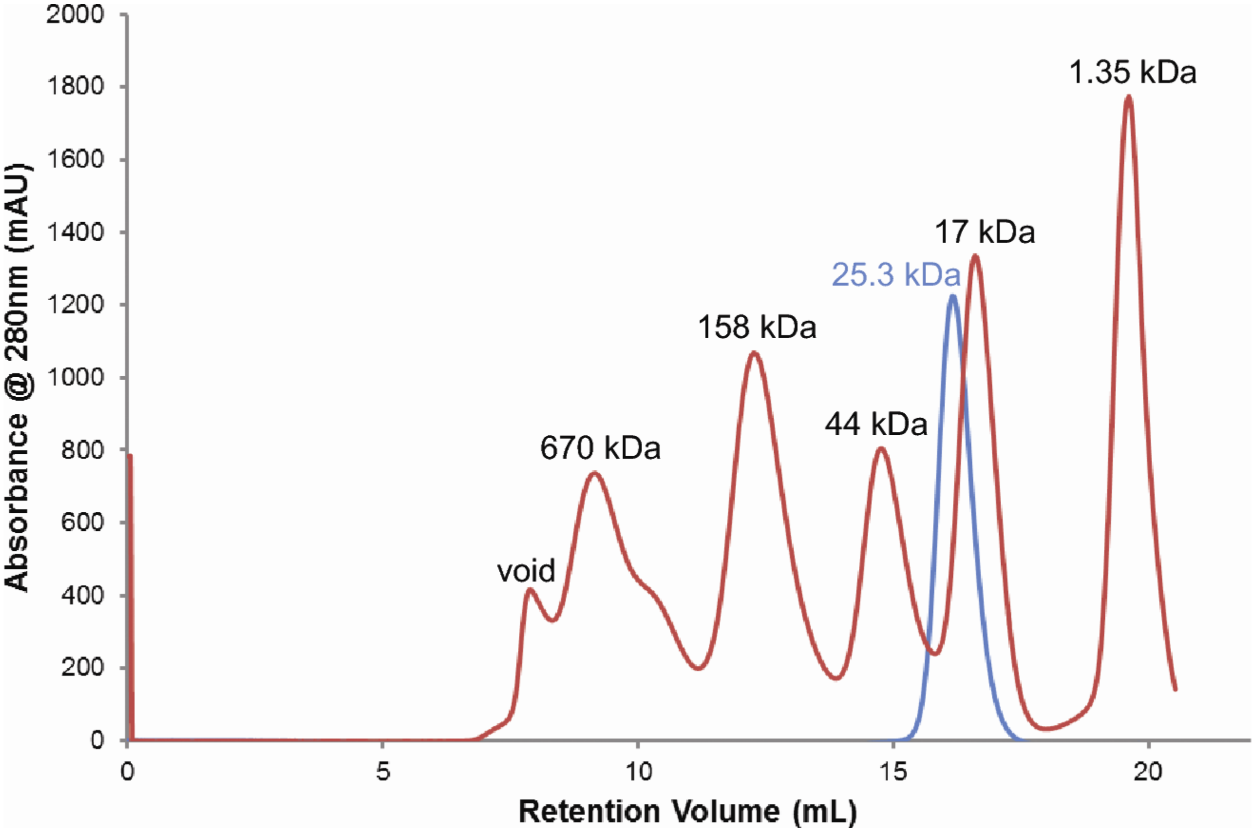

Figure S1 Determination of GBS_NucA molecular weight using size exclusion chromatography. Purified GBS_NucA (H148A) (blue) was run over a Superdex200 10/30 size exclusion column, and compared against a similar run of the BioRad Gel Filtration Standard mixture (red). The molecular weights of the protein standards are noted above each respective peak, and the actual molecular weight of the GBS_NucA (H148A) is shown in blue.

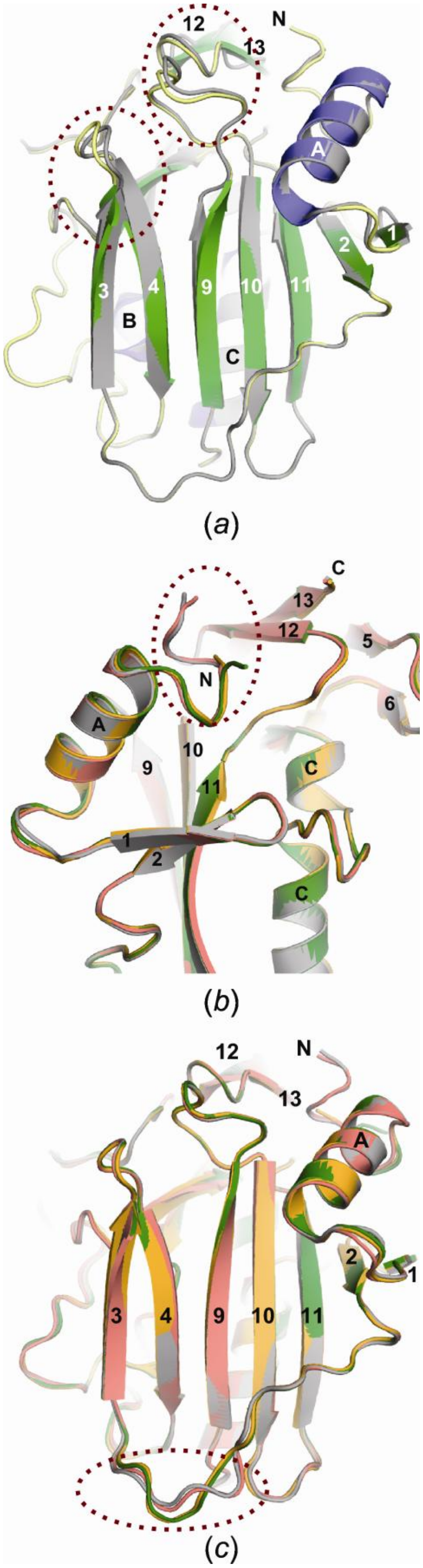
12
N
13
A
1
2
3
4
9
10
11
B
C
(*a*)
C
13
12
5
N
6
A
10
9
11
C
1
2
C
(*b*)
12
N
13
A
2
1
3
4
9
10
11
(*c*)

Figure S2 Comparison of NucA (H148A) structures from different crystal forms. (*a*) Superposition of the two NucA (H148A) apoprotein molecules per asymmetric unit (space group P1), emphasizing the 'back' face of the enzyme. Molecule A is drawn with the α-helices in blue, β-strands in green, and loop regions in yellow. Molecule B is shown in gray. (*b*) Different conformations of the N-termini of the four Mg^{2+}-bound GBS_NucA molecules per asymmetric unit in the $P6_3$ space group. Molecules A, B, C, and D are shown in green, orange, pink, and gray, respectively. (*c*) Superposition of the four NucA (H148A) molecules per asymmetric unit in space group $P6_3$, emphasizing the coil traversing the 'back' face of the enzyme. Molecules are colored as in (*b*). Slight structural variations between the molecules are indicated by red-dashed circles.

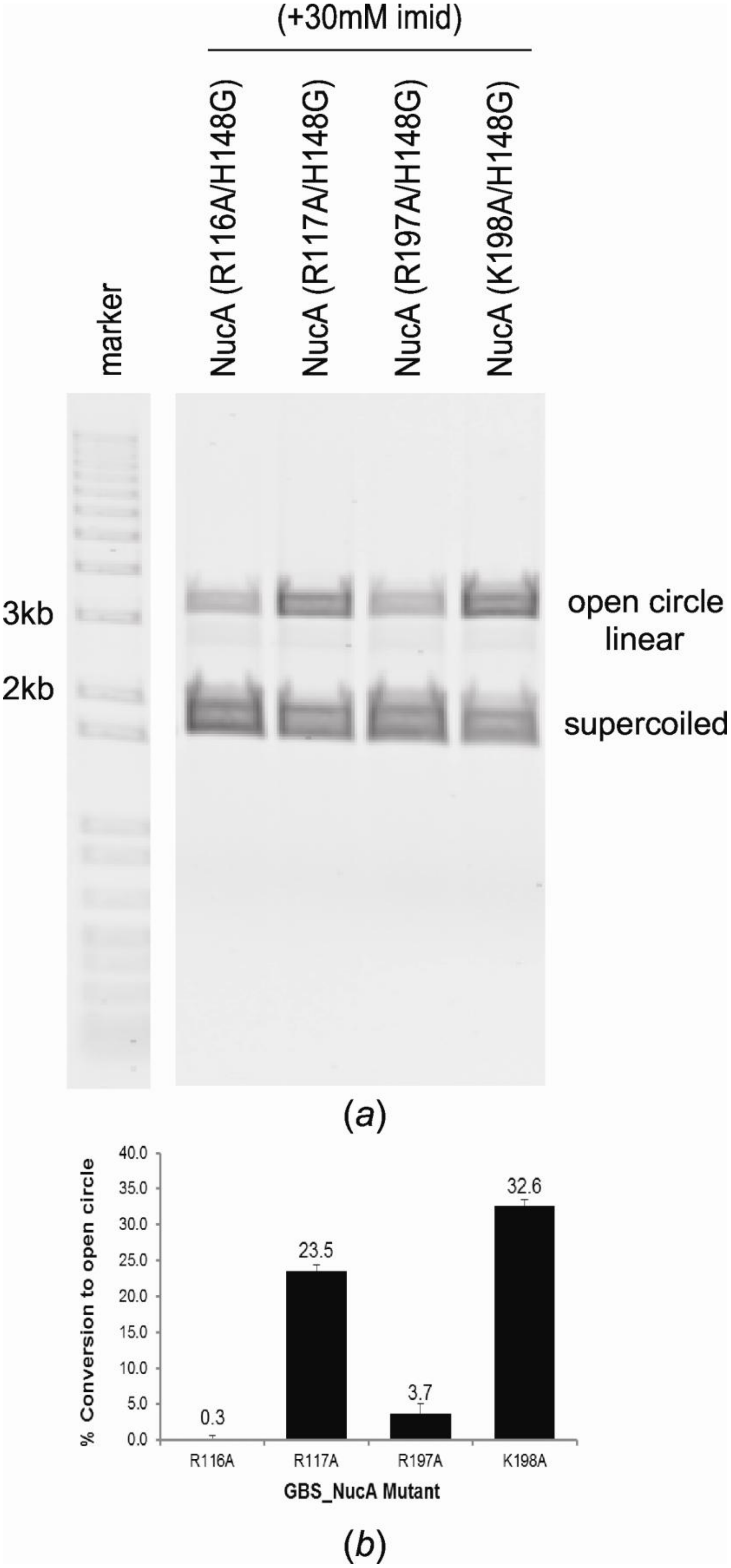
(+30mM imid)
marker
NucA (R116A/H148G)
NucA (R117A/H148G)
NucA (R197A/H148G)
NucA (K198A/H148G)
3kb
2kb
open circle
linear
supercoiled
(a)
% Conversion to open circle
40.0
35.0
30.0
25.0
20.0
15.0
10.0
5.0
0.0
0.3
23.5
3.7
32.6
R116A
R117A
R197A
K198A
GBS_NucA Mutant
(b)

Figure S3 Comparison of individual mutations in plasmid conversion nuclease activity assay. Individual mutants (R116A, R117A, R197A, and K198A) were generated on the H148G background in order to determine their role within the double mutation scenario (R116A/R117A and R197A/K198A). These mutants were then screened for catalytic activity using the imidazole chemical rescue strategy in the plasmid conversion assay (*a*). The gels were scanned using a Typhoon 9400 phosphorimager, and quantitated with ImageQuant TL (GE Healthcare). (*b*) The amount of conversion of the supercoiled plasmid substrate to the open circle form for each mutant. No further conversion to the linear form, or to lower molecular weight degraded fragments was observed. All assays were performed in triplicate, and the error bars represent the standard deviation for substrate conversion for each mutant.

Table S1 Superposition of the four molecules within the asymmetric unit of NucA (H148A) in space group $P6_3$

Values shown are the Root Mean Square Deviation of a global alignment of each molecule (Å), over a given number of Cα atoms. Superpositions and RMSD calculations were performed in COOT (Emsley & Cowtan, 2004; Emsley *et al.*, 2010)

	molA	molB	molC	molD
molA	xxx	0.185 Å over 218 Cα	0.410 Å over 212 Cα	0.445 Å over 210 Cα
molB	0.185 Å over 218 Cα	xxx	0.402 Å over 212 Cα	0.439 Å over 211 Cα
molC	0.410 Å over 212 Cα	0.402 Å over 212 Cα	xxx	0.316 Å over 214 Cα
molD	0.445 Å over 210 Cα	0.439 Å over 211 Cα	0.316 Å over 214 Cα	xxx

Supplementary references

Arnold, K., Bordoli, L., Kopp, J. & Schwede, T. (2006). *Bioinformatics* 22, 195-201.
Emsley, P. & Cowtan, K. (2004). *Acta Crystallogr D Biol Crystallogr* 60, 2126-2132.
Emsley, P., Lohkamp, B., Scott, W. G. & Cowtan, K. (2010). *Acta Crystallogr D Biol Crystallogr* 66, 486-501.
Korczynska, J. E., Turkenburg, J. P. & Taylor, E. J. (2012). *Nucleic Acids Res* 40, 928-938.
Lovell, S. C., Davis, I. W., Arendall, W. B., 3rd, de Bakker, P. I., Word, J. M., Prisant, M. G., Richardson, J. S. & Richardson, D. C. (2003). *Proteins* 50, 437-450.
